# Soil–Plant Interactions Contribute to the Mineral Composition, Nutritional Quality, and Bioactive Potential of Wild *Salicornia europaea* L. Populations in Solonchaks of the Bulgarian Black Sea Coast

**DOI:** 10.3390/plants15182861

**Published:** 2026-09-18

**Authors:** Zornitsa Zherkova, Mima Todorova, Neli Memdueva, Milena Tzanova, Neli Grozeva

**Affiliations:** 1 Department of Biological Sciences, Faculty of Agriculture, Trakia University, 6000 Stara Zagora, Bulgaria; neli.memdueva.21@trakia-uni.bg (N.M.); milena.tsanova@trakia-uni.bg (M.T.); n.grozeva@trakia-uni.bg (N.G.); 2 Department of Plant Breeding, Faculty of Agriculture, Trakia University, 6000 Stara Zagora, Bulgaria; mima.todorova@trakia-uni.bg

**Keywords:** antioxidant activity, biosaline agriculture, Bulgarian Black Sea coast, halophytes, mineral composition, nutritional value, *Salicornia europaea* L., saline soils, salinity tolerance, Solonchaks

## Abstract

Halophytes are valuable models for understanding plant adaptation to saline environments and for developing sustainable crops under increasing soil salinization and freshwater scarcity. *Salicornia europaea* L. is a highly salt-tolerant edible euhalophyte with considerable nutritional and functional potential. However, the influence of natural variability in Solonchak soils on the nutritional composition, mineral profile, and phytochemical characteristics of wild populations remains insufficiently understood. This study investigated the nutritional composition, mineral profile, antioxidant potential, oxalate content, and soil–plant relationships of six wild *S. europaea* populations growing on coastal Solonchak soils along the Bulgarian Black Sea coast. The studied soils were characterized by high electrical conductivity (up to 42.10 mS/cm) and strongly alkaline conditions (pH > 9.0). The aboveground biomass was characterized by high ash (48.49 ± 2.46%) and protein (12.91 ± 1.67%) contents, low fiber (6.02 ± 1.11%) and fat (1.03 ± 0.11%) contents, and total oxalates (11.26 ± 3.2 g/100 g), all on a dry weight basis. Na was the predominant mineral (13,866 ± 937 mg/100 g DW), followed by K, Mg, Ca, P, and Fe. The studied populations exhibited high levels of TPC, TFC, and antioxidant activity, reaching 28.6 mg GAE/g, 252.8 mg CE/g and 20.0 µmol TE/g dry weight, respectively. Correlation analysis revealed significant relationships between soil properties and plant mineral composition, oxalate content, and antioxidant traits, indicating that these characteristics are associated with multiple soil factors rather than salinity alone. These findings highlight the potential of wild *S. europaea* as a valuable source of minerals, protein, and bioactive compounds for functional foods and sustainable agriculture on saline soils.

## 1. Introduction

Halophytes represent a specialized ecological group of plants capable of completing their life cycle under saline conditions that severely restrict the growth and productivity of most glycophytic species. These plants have evolved multiple adaptive mechanisms that allow survival in salt-affected environments, including osmotic adjustment, regulation of ion transport, vacuolar sequestration of Na^+^ and Cl^−^, and accumulation of compatible organic osmolytes in the cytoplasm [1,2]. These coordinated physiological responses maintain cellular ion homeostasis, preserve metabolic activity, and enable plant growth under environmental conditions that are unsuitable for most conventional crops [3,4].

The genus *Salicornia* (Amaranthaceae) comprises highly specialized succulent halophytes widely distributed in coastal salt marshes, saline wetlands, and inland salt-affected habitats. Species of this genus have received increasing scientific attention due to their exceptional salt tolerance, ecological importance, and potential applications in saline agriculture and food production [5,6]. Moreover, Salicornia species are regarded as bioindicators of saline soils, suitable candidates for the phytoremediation of salt-affected lands, promising crops for saline agriculture, and valuable sources of functional foods rich in bioactive compounds [7,8,9,10,11,12]. Experimental and review studies have shown that several Salicornia species achieve optimal growth and biomass accumulation at soil salinity levels of approximately 200–400 mM NaCl, whereas both physiological activity and productivity generally decline outside this range [2,13,14,15,16].

In Bulgaria, natural populations of *S. europaea* occur mainly in coastal saline habitats along the Black Sea coast, particularly around Atanasovsko Lake and Pomorie Lake. These ecosystems are predominantly associated with Solonchak soils characterized by high electrical conductivity (EC), alkaline conditions, and strong spatial variability in physicochemical properties [17,18,19,20]. Such environmental heterogeneity provides an appropriate natural system for investigating how soil characteristics influence plant mineral acquisition and elemental accumulation under field conditions.

Although *S. europaea* lacks specialized salt glands, the species exhibits remarkable salt tolerance through efficient regulation of ion uptake, intracellular compartmentalization, and osmotic adjustment. The controlled accumulation of Na^+^ and Cl^−^ in aboveground tissues contributes to maintenance of cellular water relations and enables survival under saline stress [2,3]. However, salt tolerance is not determined exclusively by plant physiological mechanisms; rather, it results from complex interactions between genotype, environmental conditions, and soil properties.

Soil characteristics such as salinity level, pH, organic matter (OM) content, and mineral availability strongly influence nutrient uptake, ion competition, and elemental distribution within plant tissues. In saline environments, excessive Na^+^ accumulation may affect the uptake and allocation of essential nutrients, thereby influencing plant growth, nutritional quality, and suitability for food applications [21]. Therefore, variation in edaphic conditions among natural habitats may result in considerable differences in the mineral profile and nutritional characteristics of populations belonging to the same halophytic species.

The increasing interest in edible halophytes is associated with the need for alternative crops capable of producing valuable biomass under conditions where conventional agriculture is constrained by soil salinization, freshwater scarcity, and climate change. *Salicornia* species are considered promising candidates for saline agriculture because they can produce biomass in salt-affected environments while providing mineral-rich edible product [5,6,22]. Consequently, understanding the environmental factors controlling mineral accumulation is essential for evaluating their nutritional value, food safety, and potential agricultural utilization.

Despite the increasing interest in *Salicornia* species, available studies on *S. europaea* have mainly focused on physiological responses to experimentally imposed salinity treatments, while field-based investigations integrating soil properties with plant mineral composition remain limited. In particular, the relationships between natural soil variability and elemental accumulation in wild *S. europaea* populations from the Bulgarian Black Sea coast have not been sufficiently investigated. This knowledge gap limits the understanding of how local environmental conditions influence the nutritional quality and ecological adaptation of this species.

Therefore, the aim of the present study was to characterize the nutritional composition and mineral profile of naturally occurring *Salicornia europaea* L. populations from saline habitats along the Bulgarian Black Sea coast and to evaluate their relationships with soil physicochemical properties. We hypothesized that differences in soil conditions among habitats regulate elemental uptake and accumulation patterns, resulting in variation in mineral composition and nutritional quality of *S. europaea* biomass.

## 2. Materials and Methods

### 2.1. Study Areas and Sampling Sites

**Figure 1:**
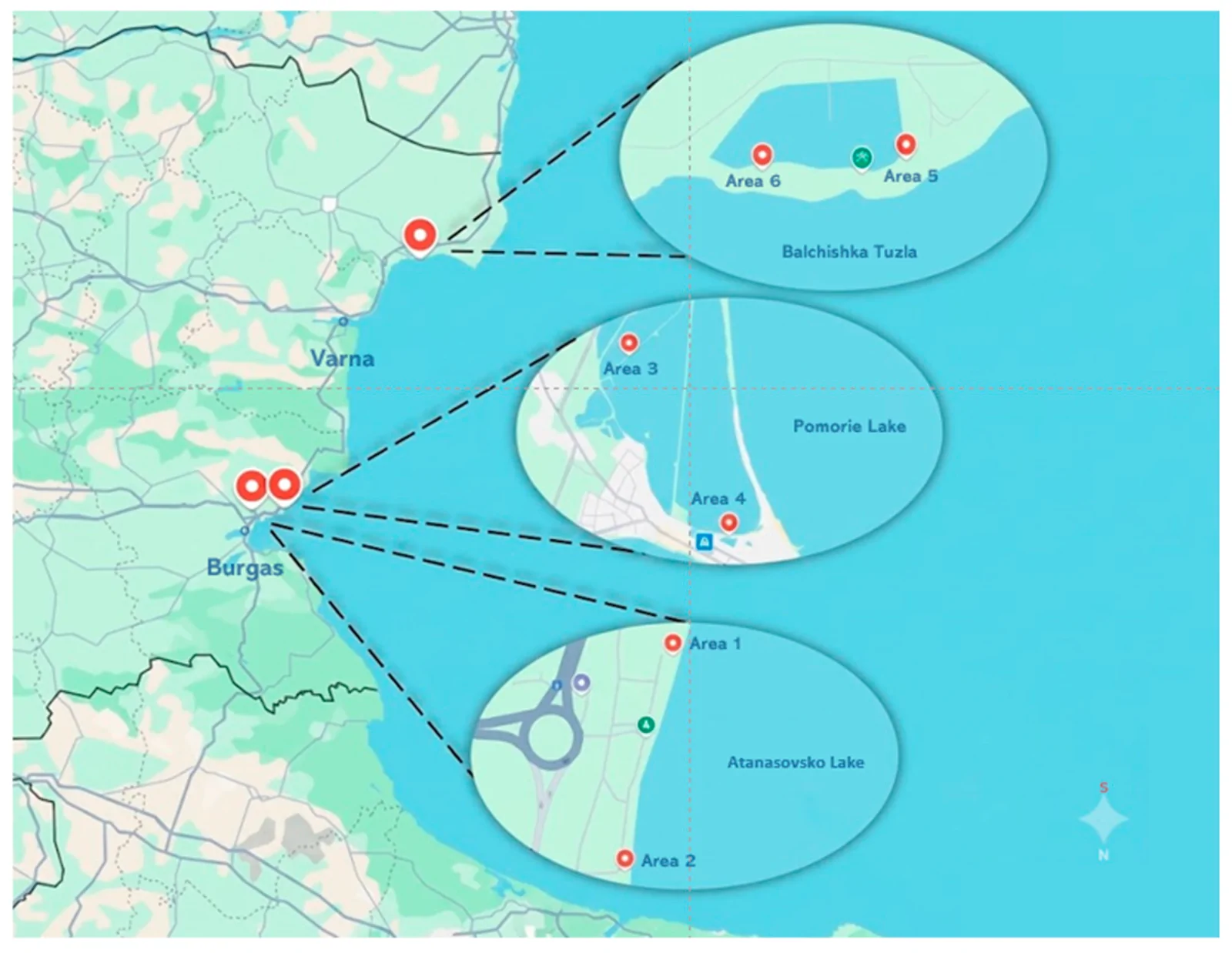
Geographic location of the investigated saline wetlands and sampling sites of *Salicornia europaea* L. populations along the Bulgarian Black Sea coast.

Aboveground biomass of *Salicornia europaea* L. and corresponding soil samples were collected during July–August 2024 from three natural saline wetlands located along the Bulgarian Black Sea coast: Atanasovsko Lake, Pomorie Lake, and Balchishka Tuzla (Figure 1). The selected sampling sites represent important natural habitats of *S. europaea* in Bulgaria and include both the northern and southern sectors of the Black Sea coastline. The study design aimed to encompass the main environmental variability associated with natural populations of the species, including differences in soil physicochemical characteristics and saline habitat conditions.

According to the World Reference Base for Soil Resources [23], the soils of all investigated sites are classified as Solonchaks. These soils are characterized by the accumulation of soluble salts within the soil profile, increased EC, alkaline soil reaction, and high concentrations of soluble and exchangeable Na^+^ and Cl^−^. Such soil properties are typical of saline environments and represent the main edaphic factors determining the occurrence and distribution of specialized halophytic vegetation [2,23].

The investigated sites are located within the coastal climatic zone of Bulgaria. Climatic conditions along the Bulgarian Black Sea coast show a transition from temperate continental influences in the northern sector to continental-Mediterranean influences in the southern sector. The maritime influence of the Black Sea reduces seasonal temperature fluctuations, resulting in mild winters, moderate summer temperatures, and a relatively extended vegetation period [24].

Atanasovsko Lake and Pomorie Lake are hypersaline coastal lagoons situated in southeastern Bulgaria, whereas Balchishka Tuzla is a shallow saline lagoon located in northeastern Bulgaria. These wetlands were formed through the interaction of marine and continental geomorphological processes and are characterized by saline soils and specific hydrological conditions supporting natural halophytic plant communities. Previous studies in the Atanasovsko Lake and Pomorie Lake regions have demonstrated that soil salinity gradients influence the distribution patterns of *Salicornia europaea* agg. and other characteristic halophytic species [19]. The sampling sites and their main characteristics are summarized in Table 1.

### 2.2. Plant and Soil Sampling

**Figure 2:**
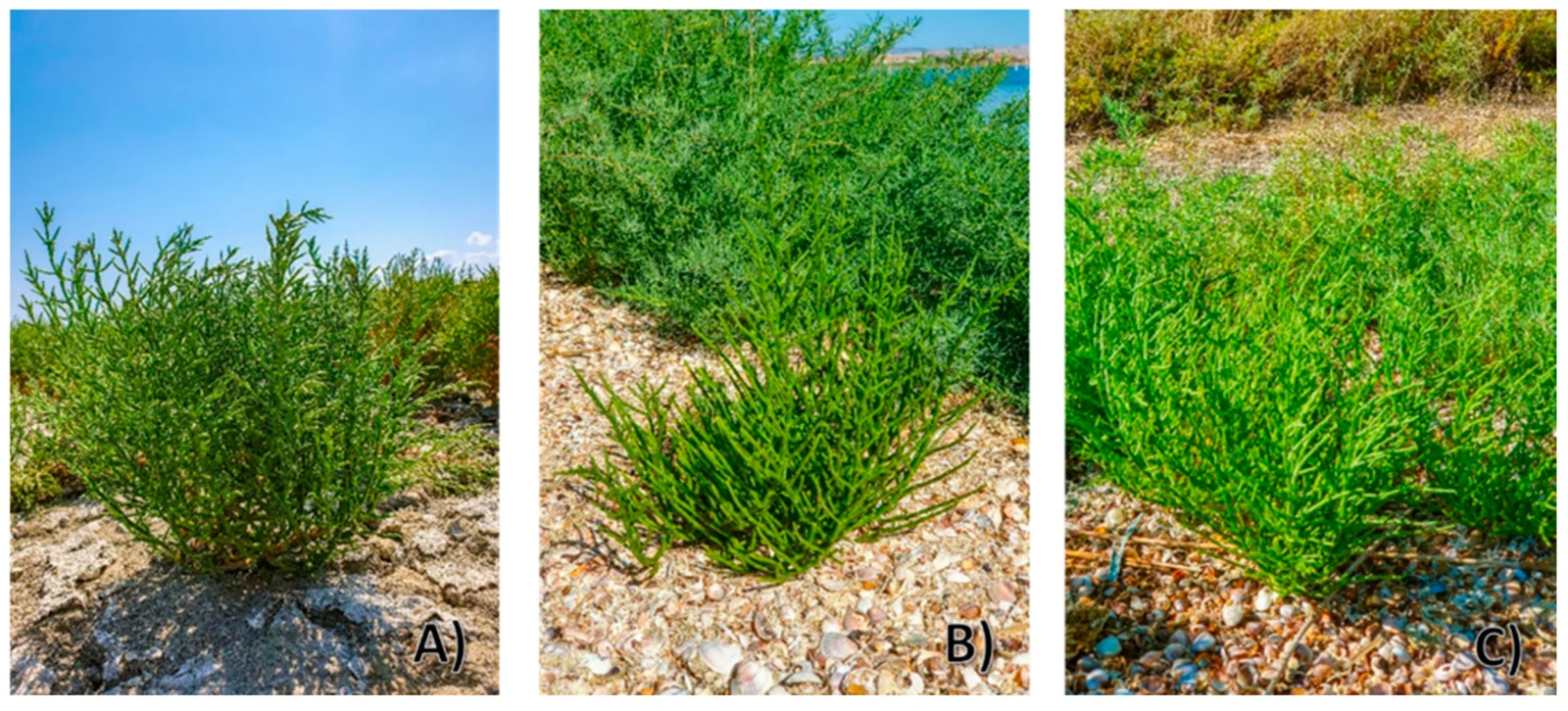
General appearance of *Salicornia europaea* L. from different natural populations along the Bulgarian Black Sea coast: (**A**) Atanasovsko Lake; (**B**) Pomorie Lake; and (**C**) Balchishka Tuzla.

Plant material was collected from naturally occurring populations of *S. europaea* using a randomized sampling design. Aboveground shoots from each sampling plot were collected at the flowering stage to minimize phenological variation in biochemical composition. The samples were immediately placed in paper bags and transported to the laboratory under cooled conditions. The general appearance of the *S. europaea* populations is shown in Figure 2.

Plant samples were collected randomly from each sampling site as composite samples, comprising approximately 1.5 kg of aboveground biomass collected from multiple individual *Salicornia europaea* plants. The collected plant material was dried at controlled temperature until constant mass was achieved, subsequently homogenized using laboratory grinding equipment, and stored under dry conditions until chemical analyses. All analytical results were calculated and expressed on a dry weight (DW) basis.

Soil samples were collected from the upper soil horizon (0–20 cm) using a stainless-steel soil auger. At each sampling site, ten soil cores were collected randomly and combined to obtain one composite soil sample representative of the local sampling area. After collection, samples were air-dried at room temperature, gently homogenized, and transported for physicochemical analysis to Lancrop Laboratories, Yara UK Ltd. (Pocklington, UK).

### 2.3. Determination of Proximate Composition

The proximate chemical composition of *S. europaea* biomass was determined according to internationally standard protocols of AOAC International, 2016, as follows:

- Moisture content was determined gravimetrically according to ISO 6496:2000 [25].
- Crude protein was calculated from total nitrogen content determined by the Kjeldahl method following ISO 5983-2:2006 [26], using the appropriate nitrogen-to-protein conversion factor.
- Crude fat was determined by solvent extraction according to ISO 6492:2007 [27].
- Crude fiber content was determined using an internally validated laboratory method (IVLM 3/2024), based on sequential acid and alkaline hydrolysis followed by gravimetric determination of the residue. Total ash content was determined gravimetrically according to ISO 5984:2002 [28].
- Nitrogen-free extract (NFE) was calculated by difference: NFE (%) = 100 − (moisture + protein + fat + fibre + ash).

### 2.4. Determination of Mineral Composition of the Plant Biomass

The mineral composition of *S. europaea* biomass was determined by an accredited analytical laboratory using microwave-assisted closed-vessel digestion with nitric acid and hydrogen peroxide, followed by inductively coupled plasma optical emission spectrometry (ICP-OES) multi-element analysis. The concentrations of Na, Ca, Mg, K, P, Mn, Zn, Fe, and Cu were determined using a PerkinElmer Optima 5300 DV instrument (PerkinElmer, Shelton, CT, USA) and expressed as mg/100 g DW. The mineral composition was determined according to ISO 22036:2024 [29].

### 2.5. Determination of Total Oxalate Content

Total oxalate (TO) concentration was determined spectrophotometrically based on oxidation of oxalate by potassium permanganate (KMnO_4_) following the protocol of Naik et al., 2014 [30]. Briefly, 0.5 g of dried and homogenized plant material was extracted with 30 mL of 0.25 N HCl at 100 °C for 15 min. The extracts were filtered and diluted to 50 mL with 0.25 N HCl.

For the analytical reaction, aliquots of extracts were mixed with sulfuric acid and potassium permanganate solution. Absorbance was measured at 528 nm on ONDA TOUCH UV-31 SCAN UV/VIS spectrophotometer (GIORGIO BORMAC SRL, Modena, Italy). TO content was determined using oxalic acid as an external standard.

### 2.6. Preparation of Methanolic Extracts and Phytochemical Analyses

Methanolic extracts were prepared using ultrasound-assisted extraction according to Tzanova et al. (2020) [31]. Briefly, dried and powdered plant material was extracted with methanol at a solid-to-solvent ratio of 1:10 (*w*/*v*). Samples were sonicated for 20 min and subsequently incubated in darkness for 24 h. Extracts were filtered through 0.45 μm membrane filters, concentrated under reduced pressure using a rotary evaporator, and reconstituted to a final concentration of 1 mg/mL.

- Total phenolic content (TPC) was determined using the Folin–Ciocalteu method, while antioxidant activity was evaluated using the 2,2-Diphenyl-1-Picrylhydrazyl (DPPH) radical scavenging assay according to Tzanova et al. (2019) [32] and Dinev et al. (2021) [33]. In brief, 1 mL sample was mixed with 5.0 mL of 1:10 *v*/*v* aqua-diluted Folin–Ciocalteu’s reagent. Then, 4 mL of 7.5% Na_2_CO_3_ was added. After 60 min, the absorbance at 765 nm was measured on ONDA TOUCH UV-31 SCAN UV/VIS spectrophotometer (GIORGIO BORMAC SRL, Modena, Italy). A calibration was established using gallic acid as a standard. The results were expressed as milligrams gallic acid equivalents (GAE) in 1 g DW.
- Total flavonoid content (TFC) was determined using a spectrophotometric aluminium chloride complexation method following protocol described by Tzanova et al. (2020) [31]. In brief, 1 mL extract, 0.3 mL 5% NaNO_3_, and 4 mL deionized water were mixed. After 5 min, 0.3 mL of 10% AlCl_3_ was added, and after another 6 min, 2 mL of 1 M NaOH was added. After homogenization, the absorbance of the solution was measured at 510 nm on ONDA TOUCH UV-31 SCAN UV/VIS spectrophotometer (GIORGIO BORMAC SRL, Modena, Italy). A calibration was established using standard solutions of catechin. The results were expressed as milligrams catechin equivalent (CE) in 1 g DW.
- Antioxidant activity was determined by DPPH radical scavenging method following the procedure described by Memdueva et al. (2026) [34]. In brief, 100 μL extract was added to 3.9 mL of 100 μM DPPH in methanol. After 30 min, the absorption at 517 nm on ONDA TOUCH UV-31 SCAN UV/VIS spectrophotometer (GIORGIO BORMAC SRL, Modena, Italy) was measured. A calibration was conducted using Trolox as a standard. The results were expressed as μmol trolox equivalent (TE) in 1 g DW.

### 2.7. Soil Physicochemical Analyses

Soil samples were analysed for pH, EC, OM, and available macro- and micronutrients. Soil pH was measured potentiometrically in a soil–water suspension. EC was determined in aqueous soil extracts (WTW conductivity meter) and was used as an indicator of soluble salt concentration. OM content was determined using the Walkley–Black dichromate oxidation method [35]. P was determined using the Olsen bicarbonate extraction method [36]. K, Ca, and Mg were determined by atomic absorption spectrometry. Micronutrients including Fe, Zn, Cu, Mn, and Mo were extracted using standard chelation procedures and quantified by AAS. All soil nutrient concentrations were expressed as mg/kg DW.

### 2.8. Statistical Analysis

Statistical analyses were performed using IBM SPSS Statistics version 26 (IBM Corp., Armonk, NY, USA). Considering the limited number of sampling areas (*n* = 6), non-parametric statistical procedures were applied. Relationships between soil physicochemical parameters and plant chemical composition were evaluated using Spearman’s rank correlation coefficient [37]. Statistical significance was defined based on the critical threshold for two-tailed tests at *n* = 6 (r_s_ ≥ 0.886 for *p* < 0.05 and r_s_ ≥ 0.943 for *p* < 0.01), with values below 0.886 treated as non-significant indicative trends. Given the exploratory nature of the study and small sample size, potential risks of Type I errors from multiple testing were acknowledged and no adjustment was applied; thus, correlations were interpreted strictly as exploratory and hypothesis-generating measures of association rather than confirmatory evidence or direct causal relationships. Soil parameters that exceeded the upper limit of quantification of the analytical method (such as soil Na, K, Mg, and S) were treated as qualitative/semi-quantitative screening data and were strictly excluded from all bivariate correlation analyses.

## 3. Results and Discussion

### 3.1. Nutritional Value and Proximate Chemical Composition of Salicornia europaea L.

The nutritional composition and metabolic profile of halophytic plants are determined by the complex interaction between species-specific adaptive mechanisms, developmental stage, and environmental conditions, particularly soil salinity and associated edaphic factors. In euhalophytic species such as *Salicornia europaea* L., these environmental drivers strongly influence ion accumulation, osmotic regulation, and biomass composition. The proximate chemical composition of the aboveground biomass collected from the studied natural populations along the Bulgarian Black Sea coast is presented in Table 2.

#### 3.1.1. Crude Ash Content

The analyzed populations exhibited exceptionally high crude ash content, ranging from 43.30% (Area 2) to 51.21% (Area 3), with an average value of 48.49 ± 2.46% DW. These values are considerably higher than those reported for cultivated *S. europaea* under controlled conditions, where ash content of approximately 31.24% was recorded [38]. Similarly, lower mineral fractions have been reported for other edible halophytes, including *Arthrocnemum macrostachyum*, *Sarcocornia perennis* subsp. *perennis*, *S. perennis* subsp. *alpini*, and *Salicornia ramosissima* [39]. The elevated ash content observed in the present study reflects the characteristic ion accumulation strategy of *S. europaea*, which is classified as a euhalophyte. Unlike salt-excreting halophytes, this species maintains osmotic balance mainly through the uptake and intracellular compartmentalization of inorganic ions, particularly Na^+^ and Cl^−^, within vacuoles. This mechanism enables the maintenance of cellular water status and metabolic activity under highly saline conditions [2,3]. Therefore, the high mineral fraction detected in the studied populations can be considered a direct consequence of the strong soil–plant interaction characteristic of saline habitats.

#### 3.1.2. Nitrogen-Free Extract (NFE)

NEE represented the second largest fraction of the biomass composition, with values ranging from 29.80% to 33.40% DW and an average concentration of 31.55 ± 1.18%. This fraction mainly includes soluble carbohydrates, which contribute to carbon storage and osmotic adjustment under saline stress conditions. The relatively stable NFE values among the investigated populations suggest a conserved metabolic response of *S. europaea* to contrasting coastal saline environments. Maintenance of carbohydrate pools is considered an important adaptive mechanism in halophytes, providing both metabolic energy and osmoprotective functions during periods of osmotic stress [4]. Differences between reported carbohydrate concentrations among studies may be associated with environmental variability, cultivation conditions, and plant developmental stage. Previous investigations have demonstrated that biochemical composition of *S. europaea* is strongly affected by growth conditions and stress intensity [38].

#### 3.1.3. Crude Protein Content

The crude protein content of *S. europaea* biomass varied from 10.33% (Area 5) to 15.11% (Area 2), with a mean value of 12.91 ± 1.67% DW. The obtained protein concentrations are comparable with previous findings for cultivated *S. europaea*, where values of approximately 13.82% were reported [38]. In comparison with related edible halophytes, the studied populations showed relatively high protein levels, confirming the nutritional potential of this species as an alternative plant-based food resource [39]. Protein accumulation in halophytes is influenced by several factors, including salinity level, nutrient availability, and ontogenetic stage. Recent studies have demonstrated that the biochemical composition of *S. europaea* changes considerably during plant development, with younger tissues generally exhibiting higher nutritional quality and increased protein concentration [40]. Therefore, the moderate variability observed among the Bulgarian populations may reflect differences in local environmental conditions and phenological status at the time of sampling.

#### 3.1.4. Crude Fiber Content

The crude fiber concentration was relatively low, ranging from 4.77% (Area 1) to 7.98% (Area 5), with an average value of 6.02 ± 1.11% DW. These values are lower than those previously reported for cultivated *S. europaea* (14.39%) [38] and for several related halophytic species [39]. The lower fiber content observed in the present study may indicate reduced structural carbohydrate accumulation in the sampling period and could contribute to the suitability of young *S. europaea* shoots as an edible vegetable resource. Variation in fiber concentration among populations may be related to differences in growth stage, environmental stress intensity, and tissue structural development. Such variability highlights the importance of considering ecological origin when evaluating the nutritional quality of wild edible halophytes.

#### 3.1.5. Crude Fat Content

The lipid fraction represented the smallest component of the proximate composition, ranging from 0.87% (Area 3) to 1.24% (Area 2), with an average value of 1.03 ± 0.11% DW.

These concentrations are consistent with previously reported values for *S. europaea* and other related halophytes [38,39]. The relatively low lipid content is typical for succulent halophytic species, where metabolic resources are preferentially allocated to osmotic regulation, ion homeostasis, and stress adaptation rather than lipid storage. Differences in lipid accumulation among populations may result from genotype–environment interactions, particularly variations in temperature, water availability, and salinity intensity. Environmental stress conditions can modify lipid metabolism by affecting membrane stabilization processes and cellular adaptation mechanisms [4].

Overall, the proximate composition of naturally occurring *S. europaea* populations from Bulgarian coastal saline wetlands demonstrates the typical nutritional profile of edible halophytes, characterized by a high mineral fraction, moderate protein content, stable carbohydrate accumulation, and low lipid levels. The observed variability among sampling sites indicates that local environmental conditions may influence biomass composition and nutritional quality. These findings support the potential of *S. europaea* as a valuable edible halophyte and highlight the importance of investigating soil–plant relationships to better understand the factors controlling mineral accumulation and nutritional characteristics in natural populations.

### 3.2. Mineral Composition

The mineral composition of the aboveground biomass of *S. europaea* collected from natural populations along the Bulgarian Black Sea coast is presented in Table 3. Mineral accumulation in halophytic plants is determined by the complex interaction between species-specific physiological adaptations, soil physicochemical characteristics, and environmental conditions. In edible halophytes, the mineral profile is of particular importance because it influences both ecological adaptation mechanisms and their potential nutritional value for human and animal consumption.

#### 3.2.1. Macroelements

Among the analyzed elements, Na showed the highest concentration in the aboveground biomass of *S. europaea*, confirming the characteristic ion accumulation strategy of euhalophytic species. The mean Na concentration recorded in the present study was 13,866.2 ± 936.7 mg/100 g DW, with slightly higher values observed in populations from the southern Black Sea coast compared with the northern sites. However, the differences between sampling areas remained relatively limited, indicating a consistent capacity of *S. europaea* populations to accumulate Na under highly saline coastal conditions. The extremely high Na content reflects the ecological adaptation of *S. europaea* to saline habitats, where vacuolar sequestration of inorganic ions represents a major mechanism for maintaining cellular osmotic balance and sustaining physiological activity under salt stress. The values reported by Antunes et al. [42] and Barreira et al. [39] were recalculated to facilitate direct comparison with the present study. Similar patterns of Na accumulation have been reported for other edible halophytes. Antunes et al. [42] found even higher Na concentrations in two related species collected from Portuguese salt marshes, namely *Sarcocornia perennis* and *Salicornia ramosissima*, with values of 16,000 ± 120 mg/100 g DW and 17,440 ± 240 mg/100 g DW, respectively. In contrast, lower concentrations were reported by Barreira et al. [39] for the same taxa, with 6410 ± 90 mg/100 g DW in *S. perennis* and 8990 ± 50 mg/100 g DW in *S. ramosissima*. These differences highlight the influence of habitat salinity, soil characteristics, and local environmental conditions on mineral accumulation patterns in halophytes. Considerably lower Na concentrations were reported by Rahmani et al. [43] for *S. europaea* populations growing in southeastern Tunisia, reaching 899.8 ± 2.3 mg/100 g DW in Gabés and 1421.2 ± 6.2 mg/100 g DW in Medenine. The substantially higher sodium accumulation observed in Bulgarian populations may be associated with differences in soil salinity, seawater influence, hydrological regime, and sampling period. Following Na, the highest concentrations among macronutrients were recorded for K and Mg, with mean values of 1235.0 ± 103.8 mg/100 g DW and 1229.0 ± 77.6 mg/100 g DW, respectively. These elements play essential physiological roles in halophytes, including in enzyme activation, photosynthetic regulation, ionic balance, and stress adaptation. The obtained K concentrations were considerably higher than those reported by Rahmani et al. [43], who recorded only 158.4 mg/100 g DW in Gabés and 173.3 mg/100 g DW in Medenine populations of *S. europaea*. More comparable values were reported for related halophytic species from Portugal, including 892 mg/100 g DW in *S. ramosissima*, 1.03 mg/100 g DW in *Sarcocornia perennis* subsp. *alpini*, and 1390 mg/100 g DW in *S. perennis* subsp. *perennis* [39]. Similarly, the mean Mg concentration observed in Bulgarian populations was higher than values reported by Rahmani et al. [43], who found only 44.7 mg/100 g DW and 38.2 mg/100 g DW in Tunisian populations. However, the obtained values were comparable with those reported for related Portuguese halophytes, where Mg concentrations ranged between 673 and 943 mg/100 g DW [39]. The concentration of Ca ranged from 288.9 mg/100 g DW (Area 2) to 504.0 mg/100 g DW (Area 3), with an average value of 396.6 ± 82.6 mg/100 g DW. These concentrations exceeded those reported by Rahmani et al. [43], who found 151.5 mg/100 g DW and 150.9 mg/100 g DW in Tunisian populations. The observed variability may reflect differences in soil exchangeable Ca availability, ionic composition of the rhizosphere, and competitive interactions between major cations. Among the analyzed macroelements, P exhibited the highest concentration, with an average value of 191.7 ± 21.2 mg/100 g DW, ranging from 160 mg/100 g DW (Area 6) to 220 mg/100 g DW (Area 2). Phosphorus is a key element involved in energy metabolism, nucleic acid synthesis, membrane formation, and regulation of plant responses to environmental stress. The relatively high P accumulation observed in the studied populations suggests efficient nutrient acquisition under saline soil conditions.

Overall, the macronutrient profile of Bulgarian *S. europaea* populations demonstrates a typical halophytic mineral signature dominated by Na, followed by K, Mg, and Ca. The high accumulation of these elements confirms the strong adaptation of the species to saline environments and indicates its potential nutritional importance, although the high sodium content should be considered when evaluating dietary applications. However, the exceptionally high sodium concentration (13,866.2 ± 936.7) requires cautious interpretation when considering *S. europaea* as a food source. Since all values are reported on a dry weight basis, realistic fresh weight serving sizes and individual daily sodium limits must be taken into account to prevent excessive dietary sodium consumption. Consequently, *S. europaea* should be viewed primarily as a specialty salt-substitute or functional food ingredient used in modest quantities, rather than a primary dietary mineral source.

#### 3.2.2. Microelements

The mean concentration of Fe was 8.88 ± 1.86 mg/100 g DW, with values ranging from 6.31 mg/100 g DW (Area 5) to 11.81 mg/100 g DW (Area 3). These concentrations were substantially higher than those reported by Rahmani et al. [43], who recorded only 1.32 mg/100 g DW in Gabés and 1.47 mg/100 g DW in Medenine populations of *S. europaea*. The obtained values are comparable with those found in commonly consumed green vegetables, such as spinach (7.16 mg/100 g DW) and kale (9.75 mg/100 g DW) [44], suggesting that *S. europaea* may represent a valuable dietary source of Fe. The lowest concentrations among the analyzed elements were observed for Mn, Zn, and Cu, with average values of 3.28 ± 1.08 mg/100 g DW, 2.67 ± 0.38 mg/100 g DW, and 1.05 ± 0.23 mg/100 g DW, respectively. Similar to the major elements, these trace element concentrations were higher than those reported for Tunisian populations by Rahmani et al. [43], who found Mn, Zn, and Cu concentrations of 0.34, 0.36, and 0.25 mg/100 g DW in Gabés and 0.28, 0.38, and 0.16 mg/100 g DW in Medenine, respectively.

The differences observed among geographical regions confirm that mineral accumulation in *S. europaea* is strongly influenced by local edaphic conditions, including soil salinity, nutrient availability, and ionic composition. Therefore, the mineral profile of natural populations should be considered a consequence of both species-specific halophytic adaptation and habitat-specific environmental factors. According to the recommended dietary allowances established by the Food and Nutrition Board [41], the mineral composition presented in Table 3 indicates that *S. europaea* contains notable levels of essential minerals. However, due to its exceptionally high Na concentration, any potential dietary applications should consider strategies for sodium reduction, processing, or controlled intake.

### 3.3. Antioxidant Activity and Oxalate Content of Salicornia europaea L.

#### 3.3.1. Antioxidant Activity

The phytochemical composition and antioxidant potential of *Salicornia europaea* L. collected from three natural saline habitats along the Bulgarian Black Sea coast (Atanasovsko Lake, Pomorie Lake, and Balchishka Tuzla) are presented in Table 4. The accumulation of bioactive compounds in halophytic plants is closely associated with their adaptive responses to environmental constraints, particularly high salinity. Salt stress induces excessive production of reactive oxygen species (ROS), which may cause oxidative damage to cellular structures, disturbances in metabolic processes, and accelerated senescence. To maintain cellular homeostasis, halophytes activate enzymatic and non-enzymatic antioxidant mechanisms, including the biosynthesis of phenolic compounds, flavonoids, and other secondary metabolites with ROS-scavenging activity [14,45,46,47,48,49].

The obtained results demonstrated considerable variation in TPC, TFC, and DPPH radical scavenging activity among the studied populations, indicating differences in the local environmental conditions and the physiological status of plants. The samples collected from Atanasovsko Lake (Areas 1 and 2) exhibited the highest antioxidant potential. In particular, Area 2 showed the highest TPC value (28.6 mg GAE/g DW), accompanied by elevated DPPH radical scavenging activity (19.4–20.0 µmol TE/g DW). The positive relationship between phenolic accumulation and antioxidant capacity has been widely reported in halophytic species and is considered an important component of their protective response against salinity-induced oxidative stress [45,46,47]. Phenolic compounds contribute to ROS detoxification through electron donation and radical stabilization, thereby protecting cellular components under stressful environmental conditions.

The high TFC observed in the investigated populations (mean value: 232.7 mg CE/g DW) further confirms the biochemical plasticity and nutraceutical potential of *S. europaea*. Differences between the present results and previously reported values for *Salicornia* and related halophyte species may be attributed to several factors, including species identity, developmental stage, extraction procedure, environmental conditions, and soil salinity regime [39,46,50]. The pronounced accumulation of phenolic compounds observed in plants originating from highly saline habitats supports the concept that secondary metabolism plays a key role in the ecological adaptation of halophytes.

#### 3.3.2. Total Oxalate Content

Oxalates are widespread plant secondary metabolites that participate in mineral regulation, ionic balance, and defense mechanisms. Oxalic acid forms soluble and insoluble complexes with mineral elements such as Ca, Fe, Na, K, and Mg, which may influence their bioavailability. Excessive accumulation of insoluble oxalates, particularly calcium oxalate crystals, may contribute to mineral immobilization and has been associated with the formation of kidney stones in susceptible individuals [51,52,53].

In the present study, TO concentrations in *S. europaea* ranged from 7.5 to 15.8 g/100 g DW, with an average value of 11.3 ± 3.2 g/100 g DW. These levels are considerably lower than those reported for several commonly consumed oxalate-rich vegetables. For comparison, *Portulaca oleracea* L. may accumulate approximately 43.3 g/100 g DW [54], while spinach (*Spinacia oleracea* L.) has been reported to contain approximately 40.1 g/100 g DW [55]. Although oxalates are often considered antinutritional compounds, they also perform important physiological functions in plants, including calcium regulation, metal homeostasis, protection against herbivory, and adaptation to abiotic stress conditions [56,57]. Furthermore, oxalate metabolism may be associated with oxidative regulation through interactions with ROS-generating and ROS-scavenging processes [58]. The highest TO concentrations were detected in plants collected from Atanasovsko Lake (Areas 1—14.9 and Areas 2—15.8 g/100 g DW). These populations also exhibited the highest TPC, TFC, and DPPH values, suggesting that oxalate accumulation may represent an integrated component of the stress-adaptation strategy of *S. europaea*. Considering the moderate oxalate levels compared with other edible plant species, the studied populations can be regarded as a valuable source of antioxidant compounds with potential nutritional applications and without the excessive oxalate accumulation characteristic of some leafy vegetables. Overall, the obtained results demonstrate that natural populations of *S. europaea* from Bulgarian saline wetlands possess significant phytochemical potential. The observed differences among habitats highlight the importance of local environmental conditions in regulating secondary metabolism and antioxidant defence mechanisms in halophytic plants. However, a limitation of the present evaluation is that only total oxalate content was determined, without differentiating between soluble and insoluble oxalate forms, which critically influence mineral bioavailability and toxicity. Consequently, total oxalate levels alone cannot directly establish complete food safety. Additionally, the effects of culinary processing or cooking methods (such as boiling or leaching) on reducing oxalate levels were not evaluated in this study and warrant future research.

### 3.4. Soil Characteristics

#### 3.4.1. Soil Physicochemical Properties (OM, pH, EC, CEC)

The physicochemical characteristics of the soils collected from the natural habitats of *Salicornia europaea* along the Bulgarian Black Sea coast are summarized in Table 5. All investigated soils are classified as Solonchaks according to the World Reference Base for Soil Resources [23] and exhibit the characteristic properties of coastal saline ecosystems, including strong alkalinity, elevated salinity, and considerable spatial heterogeneity. These edaphic conditions are primarily determined by the interaction between marine influence, hydrological regime, sediment deposition, and local geomorphological features, resulting in marked differences among the investigated wetlands.

The studied habitats displayed substantial variation in soil physicochemical properties, indicating differences in soil development and environmental conditions despite their common classification as coastal saline wetlands. Such variability is expected in Solonchak ecosystems and plays a fundamental role in regulating nutrient availability, ion mobility, and elemental uptake by halophytic vegetation [59,60]. Consequently, the spatial heterogeneity observed among the investigated sites is likely to contribute to differences in the nutritional and mineral composition of natural *S. europaea* populations.

OM content varied from 1.6 to 6.4%, with a mean value of 3.9 ± 1.9%. These values are partly higher than those generally reported for saline soils in Bulgaria, where OM typically ranges between 0.5 and 3.0% [59]. The highest OM contents were recorded at Atanasovsko Lake (Areas 1 and 2), whereas substantially lower values were observed at Pomorie Lake and Balchishka Tuzla. The relatively high accumulation of OM in Atanasovsko Lake may reflect local hydrological conditions, greater plant productivity, and reduced decomposition rates under periodically waterlogged conditions.

Soil pH ranged from 8.8 to 9.8, with a mean value of 9.4 ± 0.38, confirming the strongly alkaline character of all investigated habitats. The lowest pH values were recorded at Balchishka Tuzla, whereas Atanasovsko Lake and Pomorie Lake exhibited the highest alkalinity. These findings are consistent with the physicochemical characteristics of Bulgarian Solonchak soils described by Yolov and Kavrakov [59] and Hristov [60]. Furthermore, Todorova et al. [19] demonstrated that *Salicornia europaea* along the Bulgarian Black Sea coast is predominantly associated with habitats exhibiting pH values above 8.1, unlike other halophytes such as *Bassia hirsuta*, *Petrosimonia brachyata*, and *Salsola soda*. The consistently high soil pH therefore represents one of the principal ecological factors controlling the occurrence and competitive success of *S. europaea* in coastal saline environments.

EC varied considerably among the studied sites, ranging from 6.10 to 42.10 mS/cm, with an average value of 19.8 ± 14.5 mS/cm. The highest EC values were recorded at Atanasovsko Lake, indicating substantially greater concentrations of soluble salts compared with Pomorie Lake and Balchishka Tuzla. Differences between sampling sites within the same locality were relatively small, whereas pronounced variation occurred among wetlands, reflecting local hydrological conditions and salt accumulation processes. The measured EC values fall within the ecological range previously reported for natural populations of *Salicornia*. According to Todorova et al. [19], *Salicornia* species occur across soils with EC values between 2 and 44 mS/cm, while the highest abundance is generally associated with salinity levels exceeding 14 mS/cm. Similarly, Ungar et al. [61] reported EC values ranging from 18.9 to 142.8 mS/cm in saline habitats dominated by *Salicornia* species. The present results therefore confirm the exceptional adaptation of *S. europaea* to highly saline environments and further emphasize soil salinity as the major ecological factor determining the distribution and performance of this obligate halophyte.

#### 3.4.2. Soil Physical Properties and Texture

The physical characteristics and particle-size distribution of the investigated soils are presented in Table 6. Although all sampling sites are classified as Solonchaks, the soils exhibited considerable spatial heterogeneity in texture, reflecting differences in sedimentary origin, geomorphological evolution, and depositional processes among the studied coastal saline wetlands.

Sand was the dominant particle-size fraction, ranging from 43.98% (Area 1, Atanasovsko Lake) to 91.9% (Area 4, Pomorie Lake), with a mean value of 59.9 ± 16.0%. Silt content varied from 6.9% to 41.1% (mean 29.4 ± 11.3%), whereas clay represented the smallest fraction, ranging between 1.2% and 14.3% (mean 10.6 ± 4.8%). According to the USDA textural classification, the investigated soils were classified as Sandy (Sa), Sandy Loam (SaLo), and Sandy Silt Loam (SaSiLo), indicating the predominance of coarse-to-medium-textured substrates.

Marked differences in soil texture were accompanied by corresponding variation in soil OM content. The highest proportion of sand was recorded in Area 4 (Pomorie Lake), where the lowest OM content (1.6%) was also observed. This inverse relationship agrees with the well-established influence of soil texture on organic carbon stabilization. Coarse-textured soils possess a relatively low specific surface area and weak organo-mineral interactions, reducing their capacity to retain and physically protect OM from microbial decomposition. In addition, their high permeability and efficient aeration promote rapid mineralization of organic residues, resulting in lower soil organic carbon accumulation [62]. Conversely, soils containing higher proportions of silt and clay, particularly those from Atanasovsko Lake, exhibited greater OM contents, highlighting the important role of fine mineral fractions in the stabilization and long-term preservation of organic carbon soil through organo-mineral associations [63,64]. From a pedological perspective, the predominance of Sa and SaLo textures is characteristic of coastal Solonchaks developed on marine and lagoon sediments. Such soils generally exhibit high porosity, rapid infiltration, efficient aeration, low-to-moderate water-holding capacity and an increased susceptibility to nutrient leaching [23,65]. Under coastal saline conditions, however, these limitations are partly compensated by seawater intrusion, capillary rise of saline groundwater and continuous accumulation of soluble salts within the surface horizons, which maintain elevated ionic concentrations in the rhizosphere.

Overall, the combination of coarse texture, high salinity, alkaline soil reaction and moderate OM content create a distinctive edaphic environment that strongly influences soil water availability, nutrient dynamics and ion mobility. These physicochemical characteristics represent major ecological filters governing the distribution, persistence and competitive dominance of *S. europaea* in the natural saline habitats along the Bulgarian Black Sea coast.

#### 3.4.3. Soil Chemical Properties and Nutrient Availability

The concentrations of macro- and micronutrients determined in the investigated soils are presented in Table 7. Although all sampling sites were classified as coastal Solonchaks, their chemical composition exhibited considerable spatial variability, reflecting differences in sediment origin, hydrological conditions, marine influence, and local pedogenetic processes. Such geochemical heterogeneity is expected to influence nutrient availability, ionic interactions within the rhizosphere, and ultimately the mineral nutrition and physiological performance of *S. europaea*.

The chemical environment of all investigated soils was dominated by soluble base cations. Concentrations of Na and Mg exceeded the upper quantification limits of the analytical method (>500 and >600 ppm, respectively) in every sample, confirming the extreme salinity of the studied habitats. Together with the highly alkaline soil reaction and elevated EC, these results characterize the soils as typical coastal Solonchaks, where soluble salts constitute the principal environmental factor controlling plant establishment and community composition [2,3]. Among the remaining macronutrients, Ca concentrations ranged from 1336 to 2452 ppm (mean 1924 ± 370 ppm). The lowest concentrations were recorded at the Pomorie Lake sites, whereas soils from Atanasovsko Lake and Balchishka Tuzla contained consistently higher Ca levels. K generally exceeded 750 ppm, with lower concentrations observed only in Areas 4 and 5. The simultaneous occurrence of elevated Na, Mg, Ca, and K concentrations reflects the marine origin of these saline soils and the continuous accumulation of soluble salts within the surface horizon. Such ionic conditions strongly influence osmotic adjustment, ion competition and selective nutrient uptake by halophytic species [2,3,66]. The detected trace elements showed considerable spatial variation among the studied soils, although the magnitude of differences varied depending on the element. Fe showed the widest concentration range (21–684 ppm), with both the highest and one of the lowest values recorded within the Pomorie Lake locality, suggesting pronounced spatial variability within the studied habitats. Similar variability was observed for B (16.7–100.3 ppm) and Cu (1.8–14.7 ppm). In contrast, Mn (54.5 ± 6.8 ppm) and Zn (3.5 ± 0.9 ppm) displayed relatively limited variation among sampling sites. P ranged from 11 to 82 ppm (mean 35.5 ± 26.2 ppm), indicating differences in P availability despite the similar pedological classification of the investigated soils. Mo concentrations ranged from 0.04 to 0.31 ppm, indicating variability among the sampling sites. S concentrations exceeded the analytical reporting limit (>140 ppm) in all samples and therefore could not be quantitatively compared among sampling sites.

Overall, the investigated soils were characterized by high salinity and pronounced spatial heterogeneity in nutrient composition. Variations in the distribution of macro- and micronutrients may affect nutrient availability and ionic conditions within the rhizosphere, potentially contributing to differences in mineral accumulation and biochemical characteristics of *S. europaea* populations. Therefore, the observed variability in soil chemical properties represents an important environmental factor associated with the variation in the nutritional composition and phytochemical profile of the studied populations.

### 3.5. Correlation Between Soil and Plant Parameters

#### 3.5.1. Methodological Context and General Interpretation of Correlations

The correlation analysis presented in Table 8 identified several statistically significant relationships, as well as strong correlation trends with potential biological relevance, between soil physicochemical properties and the mineral composition and biochemical characteristics of *Salicornia europaea*. The observed relationships suggest that variation in the nutritional composition and biochemical characteristics of this euhalophyte is not determined solely by salinity level but may also be associated with other edaphic factors, including soil EC, pH, OM content, and micronutrient availability. Such relationships are increasingly recognized as characteristic of natural saline ecosystems, where plant adaptation is influenced by the combined effects of soil chemistry, rhizosphere processes, and physiological plasticity rather than by individual environmental factors acting independently [2,3,67]. Given the limited sample size (*n* = 6), the observed correlations should be interpreted with caution and regarded as indicative rather than conclusive. Nevertheless, several of the identified relationships are consistent with current knowledge of ion homeostasis, mineral nutrition, and stress adaptation in halophytes. Although these findings require confirmation through larger datasets and controlled experiments, they contribute to a better understanding of the role of soil physicochemical properties in shaping mineral accumulation and biochemical characteristics of *S. europaea* under natural saline conditions.

#### 3.5.2. Regulation of Ion Homeostasis in Relation to Soil Salinity and Organic Matter

A positive trend was observed between Na concentration in the aboveground biomass and soil EC (r_s_ = 0.829). While this non-significant trend (r_s_ ≥ 0.886) aligns with established physiological models regarding Na as an osmotic regulator in *Salicornia europaea* [2,3], it should be considered purely indicative rather than conclusive due to the limited sample size. Similarly, the negative relationships between plant K, Mg, and Ca concentrations and soil EC (r_s_ = −0.543, −0.429, and −0.371, respectively) were not statistically significant, though their negative direction is generally consistent with classical ionic antagonism described in controlled experiments [69]. In contrast, soil OM showed a statistically significant negative correlation with plant K concentration (r_s_ = −0.986, *p* < 0.05), alongside strong but non-significant negative trends in Mg (r_s_ = −0.841) and Ca (r_s_ = −0.754). These patterns suggest that soil OM is associated with reduced accumulation of these cations in the aboveground biomass, likely by influencing their mobility and bioavailability in soil [62,70,71]. Meanwhile, the weak, non-significant relationship between soil OM and plant Na concentration (r_s_ = 0.406) indicates that Na^+^ is retained less strongly by the soil sorption complex than K^+^, Ca^2+^ and Mg^2+^. This aligns with the lower affinity of monovalent Na^+^ for exchange sites compared with divalent cations. Furthermore, K^+^ may be specifically retained within clay interlayer positions owing to structural compatibility [70,71]. In this context, soil OM may act as a regulator of nutrient bioavailability through sorption and complexation, potentially creating conditions for relatively greater Na^+^ availability in the soil solution and thus possibly favoring its accumulation in aboveground biomass compared with K^+^, Ca^2+^ and Mg^2+^.

#### 3.5.3. Micronutrient Accumulation Under Saline and Alkaline Soil Conditions

In contrast to established soil chemical models, a strong positive relationship was observed between Zn concentration in the aboveground biomass and soil pH (r_s_ = 0.943, *p* < 0.05). A weaker, non-significant positive trend was also noted in Fe (r_s_ = 0.714). These patterns contrast with the classical concept that increasing soil pH reduces the solubility and bioavailability of Zn and Fe [72,73]. A similar non-significant positive trend was observed with respect to EC, where Zn concentration in the biomass showed a strong positive relationship with soil EC (r_s_ = 0.829). Although this non-significant trend deviates from soil chemical models suggesting that increasing salinity promotes the formation of aqueous Zn-chloride complexes ([ZnCl_n_]^2−n^) and reduces free Zn^2+^ ion availability [74], it should be considered indicative rather than conclusive. Altogether, these observations suggest that higher soil pH and EC tend to be accompanied by increased micronutrient levels in plant biomass, suggesting that accumulation in *S. europaea* may not be determined solely by soil chemical solubility. It is possible that specific physiological adaptations, including the release of root exudates with metal-mobilizing properties [75], combined with selective transport mechanisms and intracellular sequestration [76], contribute to maintaining Zn and Fe accumulation even under alkaline soil conditions.

#### 3.5.4. Influence of Soil Molybdenum on Cellular Metabolism

These patterns suggest that higher soil Mo concentrations are associated with the accumulation of Na and P in aboveground biomass (r_s_ = 0.812 for both; r_s_ < 0.886). In contrast, statistically significant positive correlations were identified with TO (r_s_ = 0.899, *p* < 0.05) and antioxidant activity (r_s_ = 0.941, *p* < 0.05). These patterns suggest that higher soil Mo concentrations are associated with the simultaneous enhancement of processes related to ion regulation, mineral nutrition, and the antioxidant response of *S. europaea*. Such an association is biologically plausible, as molybdenum serves as an essential cofactor for key oxidoreductases involved in nitrogen metabolism and cellular homeostasis [71,77]. Regarding phosphorus, plant phosphorus concentrations were also higher in samples from Atanasovsko Lake than in those from Balchishka Tuzla, and a positive but not statistically significant relationship (r_s_ = 0.657) was observed between soil and plant phosphorus concentrations, potentially reflecting the ability of *S. europaea* to regulate phosphorus uptake according to its metabolic needs.

#### 3.5.5. Study Limitations

Several limitations of the present study should be acknowledged. First, the evaluation is based on a limited number of sampling sites (*n* = 6) across three coastal wetlands and samples were collected during a single sampling period, which restricts statistical power and limits seasonal generalization. Second, due to the exploratory nature of the design and because no adjustment for multiple testing was applied, identified soil–plant relationships represent statistical associations intended for hypothesis generation rather than confirmed causal mechanisms. Third, direct physiological and metabolic processes (such as selective ion transport, root exudation, or antioxidant enzyme kinetics) were not measured. Finally, oxalates were quantified only as total oxalate, without differentiating between soluble and insoluble fractions. Future research incorporating larger spatial–temporal sampling and direct physiological experiments is required to validate these exploratory findings. Additionally, since the six sampling sites were distributed across three wetlands (two areas per wetland), local similarities between paired locations may exist, which should be taken into account when generalizing these findings.

## 4. Conclusions

The present study of wild *Salicornia europaea* L. populations from Atanasovsko Lake, Pomorie Lake, and Balchik Tuzla provides baseline insights into the nutritional, mineral, and phytochemical characteristics of the species in highly saline Black Sea coastal habitats. The results indicate that the aboveground biomass is characterized by high mineral content (particularly Na, K, Mg, and Ca), moderate protein, low fat and fiber, and notable in vitro phenolic and antioxidant activity, alongside relatively low oxalate levels. Correlation analysis suggests that, alongside soil salinity, edaphic factors such as pH, OM content, and micronutrient availability are associated with variations in plant composition. No statistically significant correlations were identified between soil chemical properties and primary nutritional parameters (protein, crude fat, and crude fiber). Given the limited sample size (*n* = 6) and uncorrected multiple comparisons, these bivariate relationships represent exploratory, hypothesis-generating ecological trends rather than confirmed physiological mechanisms. From a practical perspective, the findings suggest the preliminary potential of *S. europaea* as an edible halophyte for saline agriculture; however, its high sodium accumulation dictates that dietary integration should be limited to modest portion sizes (e.g., fresh garnishes) or processed biomass (e.g., dried salt substitutes or blanched vegetables). Finally, as the observed antioxidant activity represents in vitro biochemical capacity, future in vivo trials, bioaccessibility evaluations, and broader spatial sampling are necessary to substantiate any health-related claims and fully evaluate the species’ nutritional value.

## Figures and Tables

**Table 1:** The geographical coordinates, altitude, soil classification, and herbarium voucher numbers of the sampled populations.

Site ID	Location	Voucher Specimens	Coordinates	Altitude, m	Soil Type
Area 1	Atanasovsko Lake	SOA 063638	42°31′56.7″ N 27°28′18.0″ E	7	Solonchaks
Area 2		SOA 063637	42°31′45.5″ N 27°28′14.5″ E		
Area 3	Pomorie Lake	SOA 063640	42°35′08.6″ N 27°36′58.4″ E	−0.65	Solonchaks
Area 4		SOA 063639	42°33′56.3″ N 27°37′54.7″ E		
Area 5	Balchishka Tuzla	SOA 063635	43°24′00.9″ N 28°13′25.2″ E	4	Solonchaks
Area 6		SOA 063636	43°24′00.1″ N 28°13′08.0″ E		

**Table 2:** Nutritional value of *Salicornia europaea* L. from the studied areas, DW.

Site ID	Location	Crude Protein, %	Crude Fat, %	Crude Fiber, %	Ash, %	NFE, %
Area 1	Atanasovsko Lake	14.32	0.95	4.77	49.16	30.81
Area 2		15.11	1.24	6.75	43.30	33.59
Area 3	Pomorie Lake	12.42	0.87	4.86	51.21	30.64
Area 4		13.83	1.03	5.75	48.86	30.54
Area 5	Balchishka Tuzla	10.33	1.05	7.98	49.68	30.96
Area 6		11.45	1.03	6.03	48.73	32.76
Min		10.33	0.87	4.77	43.30	30.54
Max		15.11	1.24	7.98	51.21	33.59
Mean ± SD		12.91 ± 1.67	1.03 ± 0.11	6.02 ± 1.11	48.49 ± 2.46	31.55 ± 1.18

**Table 3:** Mineral composition of *Salicornia europaea* L. from the studied areas, DW.

Site ID	Location	Na, mg/100 g	Ca, mg/100 g	Mg, mg/100 g	K, mg/100 g	Mn, mg/100 g	Zn, mg/100 g	Fe, mg/100 g	Cu, mg/100 g	P, mg/100 g
Area 1	Atanasovsko Lake	14,747.6	300.9	1146.4	1099.7	5.10	3.23	9.54	1.33	210
Area 2		14,892.8	288.9	1108.0	1104.2	4.36	2.51	8.46	1.39	220
Area 3	Pomorie Lake	14,042.5	504.0	1312.0	1350.1	2.67	3.08	11.81	0.82	170
Area 4		14,307.9	487.9	1305.4	1359.7	2.14	2.68	10.13	0.89	200
Area 5	Balchishka Tuzla	12,698.1	387.3	1273.4	1256.5	2.41	2.13	6.31	0.95	190
Area 6		12,508.7	410.6	1228.6	1239.3	3.02	2.39	7.05	0.90	160
Min		12,508.6	288.9	1108.0	1099.7	2.14	2.13	6.31	0.82	160
Max		14,892.8	504.0	1312.0	1359.7	5.10	3.23	11.81	1.39	220
Mean ± SD		13,866.2 ± 936.7	396.6 ± 82.6	1229.0 ± 77.6	1234.9 ± 103.9	3.28 ± 1.08	2.67 ± 0.38	8.88 ± 1.86	1.05 ± 0.23	191.7 ± 21.2
* Recommended daily intake (adult), mg/day		1600	1200	1600	1650–1875	270–400	12–15	12–15	1.5–3	700

* Daily requirement of adult [41].

**Table 4:** Antioxidant potential and oxalate content in DW of *Salicornia europaea* L.

Site ID	Location	DPPH, µmol TE/g	TFC, mg CE/g	TPC, mg GAE/g	TO, %
Area 1	Atanasovsko Lake	20.0	241.4	21.5	14.9
Area 2		19.4	252.8	28.6	15.8
Area 3	Pomorie Lake	17.7	230.9	14.6	11.5
Area 4		17.6	227.0	14.4	10.2
Area 5	Balchishka Tuzla	17.7	220.2	13.1	7.7
Area 6		17.3	224.0	13.9	7.5
Min		17.3	220.2	13.1	7.5
Max		20.0	252.8	28.6	15.8
Mean ± SD		18.3 ± 1.1	232.7 ± 12.2	17.7 ± 6.2	11.3 ± 3.2

DPPH—2,2-diphenyl-1-picrylhydrazyl; TPC—total phenolic content; TFC—total flavonoid content; TO—total oxalate; SD—standard deviation; μmol TE/g—μmol as Trolox equivalent; mg CE/g—mg as catechin equivalent; mg GAE/g—mg as gallic acid equivalent.

**Table 5:** Soil physicochemical properties across the sampling sites.

Site ID	Location	OM, %	pH	EC, mS/cm
Area 1	Atanasovsko Lake	6.4	9.8	42.1
Area 2		6.3	9.5	37.6
Area 3	Pomorie Lake	2.6	9.7	13.5
Area 4		1.6	9.4	12.3
Area 5	Balchishka Tuzla	2.6	8.8	6.1
Area 6		3.7	8.9	7.2
Min		1.6	8.8	6.1
Max		6.4	9.8	42.1
Mean ± SD		3.9 ± 1.9	9.4 ± 0.38	19.8 ± 14.5

**Table 6:** Physicochemical properties and physical parameters across the sampling sites.

Site ID	Location	Physical Analysis			Physical Properties					
		Sand, %	Silit, %	Clay, %	Soil Type	Available Water	Drainage Rate	Inherent Fertility	Leaching Risk	Warming Rate
Area 1	Atanasovsko Lake	44.0	41.1	14.2	SaSiLo	Low to Medium	Rapid	Low to Medium	High to Moderate	Rapid
Area 2		46.0	39.8	14.2	SaSiLo	Low to Medium	Rapid	Low to Medium	High to Moderate	Rapid
Area 3	Pomorie Lake	55.1	30.6	14.3	SaLo	Low to Medium	Rapid	Low to Medium	High to Moderate	Rapid
Area 4		91.9	6.9	1.2	Sa	Very Low to Low	Very Rapid	Low to Medium	High to Moderate	Rapid
Area 5	Balchishka Tuzla	57.0	31.3	11.7	SaLo	Low to Medium	Rapid	Low to Medium	High to Moderate	Rapid
Area 6		65.5	26.7	7.7	SaLo	Low to Medium	Rapid	Low to Medium	High to Moderate	Rapid
Min		44.0	6.9	1.2						
Max		91.9	41.1	14.3						
Mean ± SD		59.9 ± 16.0	29.4 ± 11.3	10.6 ± 4.8						

Sa—Sand; SaLo—Sandy Loam; SaSiLo—Sandy Silt Loam.

**Table 7:** Contents of micro- and macroelements in the soils across the sampling sites.

Site ID	Location	ppm											
		Ca	Mg	K	Na	Mn	B	Cu	Mo	Fe	Zn	S	P
Area 1	Atanasovsko Lake	2452	>600	>750	>500	48	94.9	14.7	0.30	145	3.5	>140	82
Area 2		2041	>600	>750	>500	51	43.4	13.4	0.31	251	4.1	>140	59
Area 3	Pomorie Lake	1336	>600	>750	>500	68	100.3	14.5	0.06	684	3.1	>140	25
Area 4		1558	>600	668	>500	58	27.0	1.8	0.05	37	2.1	>140	24
Area 5	Balchishka Tuzla	2054	>600	648	>500	50	16.7	2.3	0.06	40	4.9	>140	11
Area 6		2103	>600	>750	>500	52	24.7	3.3	0.04	21	2.9	>140	12
Min		1336	-	648	-	48	16.7	1.8	0.04	21	2.1	-	11
Max		2452	-	668	-	68	100.3	14.7	0.31	684	4.9	-	82
Mean ± SD		1924 ± 370	-	658 ± 10	-	54.5 ± 6.8	51.16 ± 33.8	8.3 ± 5.9	0.14 ± 0.12	196.3 ± 232.4	3.43 ± 0.9	-	35.5 ± 26.2

**Table 8:** Spearman correlation coefficients (r_s_) between soil physicochemical parameters and nutritional, mineral, and phytochemical characteristics of the aboveground biomass of *Salicornia europaea* L. (sample size, *n* = 6).

	Soil	Mo	OM	pH	EC
Plant					
Na		0.812	0.406	0.657	0.829
Ca		−0.696	−0.754	−0.086	−0.371
Mg		−0.551	−0.841	−0.143	−0.429
K		−0.580	−0.986 **	−0.371	−0.543
Zn		0.319	0.261	0.943 **	0.829
Fe		0.058	−0.232	0.714	0.543
P		0.812	0.406	0.429	0.657
TO		0.899 *	0.493	0.771	0.886 *
DPPH		0.941 *	0.647	0.667	0.754
TFC		0.754	0.580	0.829	0.943 **
TPC		0.754	0.580	0.829	0.943 **

* The correlation is statistically significant at *p* < 0.05 (critical value r_s_ ≥ 0.886). ** The correlation is statistically significant at *p* < 0.01 (critical value r_s_ ≥ 0.943). Note: Sample size (*n* = 6), the distribution of the Spearman rank correlation coefficient requires high critical thresholds to achieve statistical significance [68]. Correlation coefficients with high absolute values, such as 0.812 and 0.829, indicate potential biological trends, but do not reach the threshold for two-tailed statistical significance. These relationships are therefore interpreted as indicative trends rather than statistically significant associations.

## Data Availability

The data presented in this study are available on request from the corresponding author.

## Abbreviations

DW	Dry Weight
TO	Total Oxalate
TPC	Total Phenolic Content
DPPH	2,2-Diphenyl-1-Picrylhydrazyl
GAE	Gallic Acid Equivalents
TFC	Total Flavonoid Content
CE	Catechin Equivalent
TE	Trolox Equivalent
EC	Electrical Conductivity
OM	Organic Matter
Sa	Sandy
SaLo	Sandy Loam
SaSiLo	Sandy Silt Loam
ICP-OES	Inductively coupled plasma optical emission spectrometry
